# Supplementary figures and images for: Analysis of the fusion of multimodal sentiment perception and physiological signals in Chinese-English cross-cultural communication: Transformer approach incorporating self-attention enhancement
Source: PeerJ Comput Sci. 2025 May 23;11:e2890. doi: 10.7717/peerj-cs.2890 (PMC12192752; doi:10.7717/peerj-cs.2890)

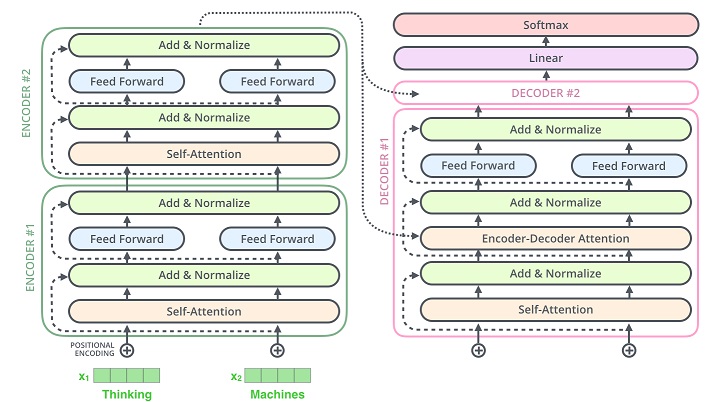

Supplement: Supplemental Information 1 [file peerj-cs-11-2890-s001.zip › Analysis of the fusion-code/image/enc_dec.jpg]

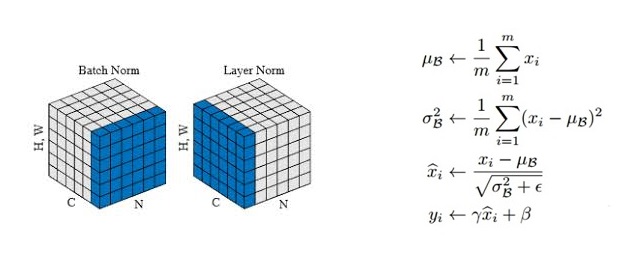

Supplement: Supplemental Information 1 [file peerj-cs-11-2890-s001.zip › Analysis of the fusion-code/image/layer_norm.jpg]

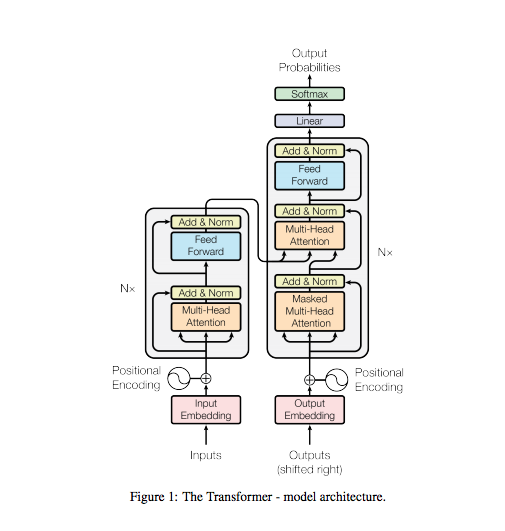

Supplement: Supplemental Information 1 [file peerj-cs-11-2890-s001.zip › Analysis of the fusion-code/image/model.png]

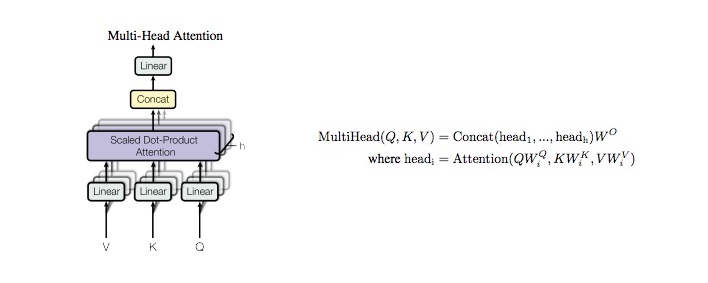

Supplement: Supplemental Information 1 [file peerj-cs-11-2890-s001.zip › Analysis of the fusion-code/image/multi_head_attention.jpg]

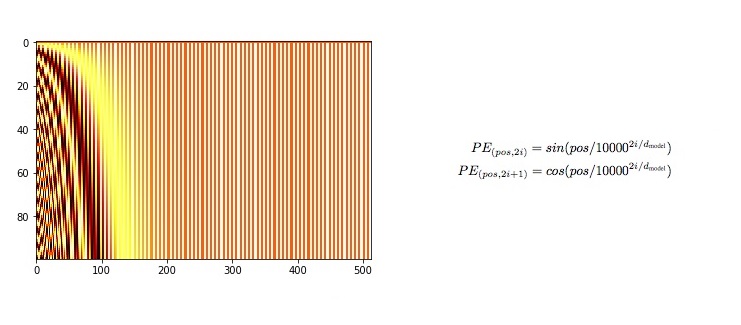

Supplement: Supplemental Information 1 [file peerj-cs-11-2890-s001.zip › Analysis of the fusion-code/image/positional_encoding.jpg]

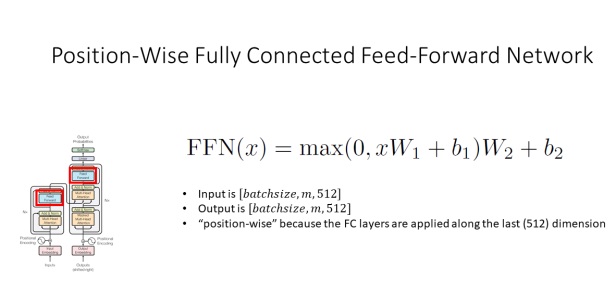

Supplement: Supplemental Information 1 [file peerj-cs-11-2890-s001.zip › Analysis of the fusion-code/image/positionwise_feed_forward.jpg]

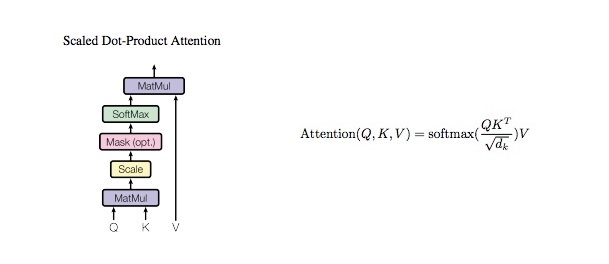

Supplement: Supplemental Information 1 [file peerj-cs-11-2890-s001.zip › Analysis of the fusion-code/image/scale_dot_product_attention.jpg]

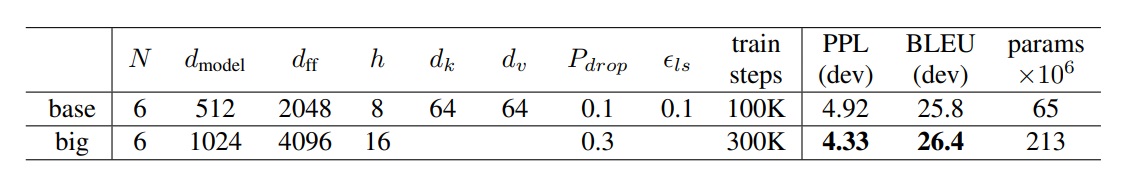

Supplement: Supplemental Information 1 [file peerj-cs-11-2890-s001.zip › Analysis of the fusion-code/image/transformer-model-size.jpg]

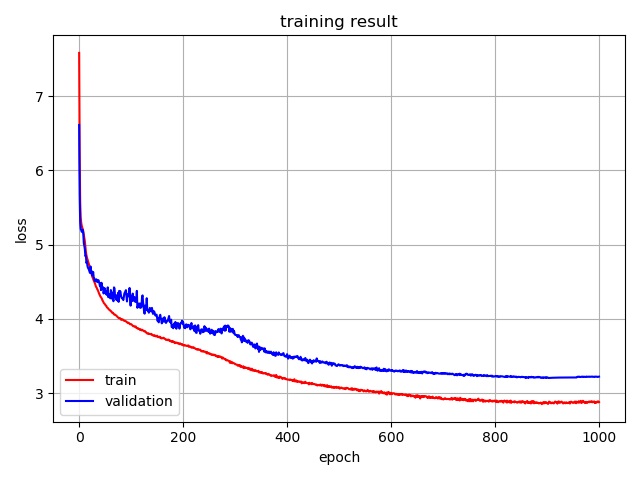

Supplement: Supplemental Information 1 [file peerj-cs-11-2890-s001.zip › Analysis of the fusion-code/saved/transformer-base/train_result.jpg]
